# Empowering Masters of Creative Problem Solvers: The Impact of STEM Professional Development Training on Teachers’ Attitudes, Self-Efficacy, and Problem-Solving Skills

**DOI:** 10.3390/jintelligence13100132

**Published:** 2025-10-20

**Authors:** Mehmet Durnali, Bayram Gökbulut

**Affiliations:** Department of Educational Sciences, Ereğli Faculty of Education, Zonguldak Bulent Ecevit University, Ereğli 67350, Zonguldak, Turkey

**Keywords:** STEM, professional development, teacher training, self-efficacy, problem solving skills

## Abstract

We aimed to explore the effects of hands-on STEM training on teachers’ attitudes toward STEM, their self-efficacy in implementing STEM methodologies, and their problem-solving skills. Additionally, we explored teachers’ professional competence in integrating STEM applications into their instructional practices and the impact of these applications on students’ 21st-century skills. The study involved 30 in-service teachers participating in a STEM training program. A nested-methods approach was adopted, combining quantitative and qualitative analyses. Pre- and post-training data were collected using Likert-type scales measuring teachers’ self-efficacy, problem-solving skills, and attitudes toward STEM. Additionally, semi-structured interviews were conducted after the training to capture teachers’ perceptions and experiences. The findings indicated significant improvements in teachers’ self-efficacy and problem-solving skills. Thematic analysis of interview data identified key factors influencing successful STEM implementation, including collaboration, technology integration, and social-emotional learning. Teachers also reported that the training positively influenced their students’ problem-solving and critical thinking skills. This study highlights the importance of professional development in enhancing teachers’ competencies for effective STEM education. The findings contribute to the literature by providing insights into teachers’ first-hand experiences and perceptions regarding the impact of STEM training on their instructional practices and student learning outcomes.

## 1. Introduction

An intricate kaleidoscope of unprecedented complex and interconnected challenges confronts humanity, calling for innovative solutions that interlace diverse disciplines together. Science, technology, engineering, and mathematics (STEM) stand tall as pillars of progress, holding the key to addressing global issues like climate change, environmental degradation, resource scarcity, and population growth (Thomas and Watters 2015). Unlocking their potential hinges on a crucial agent: teachers. In other words, teachers equipped with deep STEM knowledge, positive attitudes, and robust problem-solving skills are the architects of future generations empowered to confront these complexities.

A good deal of existing research emphasizes the transformative power of STEM education, linking it to national competitiveness, economic prosperity, and individual skill development (i.e., Kelley and Knowles 2016). To illustrate, STEM education helps bridge the gap between abstract knowledge and concrete applications. Students see how science, technology, engineering, and math concepts work in the real world (Kelley and Knowles 2016), making learning more meaningful and engaging. By integrating STEM disciplines into real-world contexts, students can acquire conceptual abilities that they need to thrive, as well as critical 21st-century skills—scientific inquiry, technological literacy, engineering design, and mathematical thinking—that empower them to become competitive, responsible, and impactful citizens (Breiner et al. 2012).

No doubt a good deal of literature will find that teachers play a pivotal role in guiding students towards STEM proficiency. Therefore, some scholars (i.e., Goldhaber et al. 2015) have highlighted that the demand for teachers’ professional development in the STEM approach has been continuously rising. In response, nations are allocating resources and implementing policies to bolster teacher professional development in STEM fields (Johnson 2013), and supra-national organizations such as the European Union are no exception. Despite these efforts, because of widespread recognition of its importance, some countries are witnessing a concerning decline in STEM education interest. Even with the adoption of student-centered approaches in Western nations, student engagement with STEM appears to be dwindling (Thomas and Watters 2015).

This decline can be partially attributed to negative teacher attitudes towards STEM. Unlike traditional application-based instruction, STEM presents unique challenges for teachers, often leading to difficulties in implementation (Huang et al. 2022). These negative attitudes often stem from professional inadequacies, insufficient subject-matter knowledge, and limited engagement in professional development activities (Holdren et al. 2010). Notably, self-efficacy plays a key role in teachers’ decisions to participate in such programs. Regular participation in professional development opportunities has been shown to positively impact teachers’ job satisfaction and self-efficacy. Furthermore, high self-efficacy perceptions in teachers translate to enhanced student belief in their own academic success (Midgley et al. 1989).

Furthermore, recent international studies have documented a clear drop in young learners’ STEM interest by late elementary/middle school. For example, one recent analysis observed that science motivation “declines typically…during the transition from primary to secondary education” (Arifin et al. 2024). Consistent with this, a UK survey found that the share of 11–14-year-olds rating science as “very or fairly interesting” fell from 76% in 2019 to 71% in 2023 (Royal Society and Engineering UK 2024). Similarly, a longitudinal study in Germany reported a marked fall in physics interest between grades 5 and 7 (Steidtmann et al. 2023). These trends have been especially noted in Western countries, where policymakers express concern over waning STEM engagement. At the same time, many primary teachers report hesitancy toward teaching STEM, often blaming inadequate training. Empirical studies show that elementary teachers commonly experience anxiety and feelings of inadequacy from their limited STEM content knowledge (Papagiannopoulou and Vaiopoulou 2024). For instance, a recent U.S. study found that most elementary teachers have “inadequate STEM background knowledge” and correspondingly low confidence; this gap in preparation was linked to reluctance to include hands-on STEM activities in their classrooms (Buechel et al. 2024; Papagiannopoulou and Vaiopoulou 2024).

By contrast, teachers’ confidence and beliefs strongly predict whether they embrace STEM instruction. A meta-analysis of STEM professional development found that high-quality training produces a large increase in teachers’ STEM self-efficacy (overall effect size g ≈ 0.64) (Zhou et al. 2023). In practice, educators with stronger STEM self-efficacy are far more likely to implement integrated STEM lessons and persist with them: one study noted that “Elementary teachers with high STEM self-efficacy adopt STEM instruction to the classroom better than others who do not have high STEM self-efficacy” (Buechel et al. 2024). Conversely, low-efficacy teachers—often those with only generalist training—tend to avoid new STEM approaches (Buechel et al. 2024). Crucially, teachers’ pedagogical beliefs also shape STEM uptake. Reviews report that teachers’ own efficacy beliefs and the value they attach to STEM significantly influence their willingness to engage with STEM curricula (Margot and Kettler 2019). In other words, educators who favor inquiry- and project-based pedagogy (aligned with STEM) generally show more enthusiasm for STEM teaching, whereas those with more traditional, teacher-led views are less inclined.

On the other hand, a growing body of literature (i.e., Honey et al. 2014; Li and Anderson 2020) has raised concerns about the overly utilitarian and economy-driven nature of many STEM education reforms, which risk reducing learning to workforce preparation rather than fostering holistic educational outcomes. Some scholars argue that STEM curricula often lack cultural and contextual relevance, particularly when applied uniformly across diverse educational systems, and may inadvertently marginalize students or educators who do not identify with dominant discourses of technological and scientific progress (i.e., Ejiwale 2013). These systemic shortcomings may partly explain why some teachers exhibit low motivation or ambivalence toward STEM: not due to resistance per se, but due to discomfort with the instrumentalist emphasis and epistemological complexity of STEM integration (Vossen et al. 2020).

Furthermore, the challenges of implementing interdisciplinary teaching—often assumed in STEM approaches—have also been criticized. Teachers, especially in primary and middle school settings, frequently lack both structural support and adequate professional development to effectively integrate science, technology, engineering, and mathematics into a coherent pedagogical framework (Margot and Kettler 2019; Thibaut et al. 2018a). When STEM education is introduced without critical reflection on curricular coherence, subject identity, or teacher agency, it can generate confusion and professional resistance, rather than the intended innovation (English 2016).

These critiques provide a valuable lens for understanding why some primary and middle school teachers demonstrate low interest or hesitancy toward STEM adoption. Rather than stemming from outright resistance, such responses may reflect legitimate concerns about the utilitarian framing of STEM, its perceived lack of cultural or curricular relevance, and the challenges of interdisciplinary integration in under-resourced school environments (Ejiwale 2013; Thibaut et al. 2018b). These systemic barriers can erode teachers’ confidence, particularly when they are not adequately trained or supported to navigate the epistemological complexity of STEM education (Vossen et al. 2020). Against this backdrop, our research problem gains further significance: we investigate how teacher self-efficacy and pedagogical beliefs mediate engagement with STEM, not only as personal traits, but as key interpretive filters through which educators make sense of broader reforms. By adopting a critically informed stance, this study acknowledges the ambivalent and context-dependent nature of STEM implementation. Rather than viewing STEM as a universally beneficial solution, we frame it as a pedagogical approach whose success depends on how well it aligns with teachers’ professional identities, belief systems, and levels of instructional confidence (English 2016; Margot and Kettler 2019).

Taken together, these findings—grounded in empirical research—and international criticism suggest that boosting teachers’ STEM knowledge and confidence can increase student engagement, replacing speculative “vicious cycle” claims with evidence that targeted support for teachers can reverse declining STEM participation. Accordingly, it is obvious that equipping students with these crucial abilities necessitates that teachers themselves possess them first. This can be achieved through their continuous participation in professional development programs focused on STEM. Building upon all research, especially on Kutnick et al.’s (2022) study, we posit that systematically developing and implementing innovative and creative STEM training for teachers is of paramount importance. Such training should specifically focus on fostering teachers’ positive attitudes towards STEM, enhancing their self-efficacy in applying STEM concepts in the classroom, and strengthening their problem-solving skills. In doing so, we can help to cultivate a generation of teachers equipped to effectively transmit knowledge, foster positive attitudes, and empower students to tackle global challenges like climate change.

To that end, with this research, we delved into the impact of a professional development program designed to foster teachers’ positive attitudes towards STEM, enhance their self-efficacy in applying STEM concepts in the classroom, and bolster their problem-solving skills. Additionally, we explored the views of teachers participating in the program’s various aspects, offering valuable insights into their experiences and perspectives. We set the following specific quantitative questions:

- Does participation in the STEM professional development program result in significant pre-test to post-test differences in teachers’ attitudes towards STEM?
- Is there statistically significant pre-test-to-post-test improvement in teachers’ self-efficacy levels regarding STEM implementation in the classroom?
- Does the program lead to demonstrably enhanced problem-solving skills among participating teachers, as measured by pre-test and post-test comparisons?

Furthermore, the following qualitative questions were designed to gain a deeper sight into the program’s effectiveness and perceived value:

- Following the training, do participating teachers perceive themselves as capable of integrating STEM applications into their lessons or classroom practices? If so, what are the reasons behind that confidence?
- In participating in teachers’ perspective, has the STEM program contributed to their professional development as a teacher? If so, how has it specifically impacted their practice?
- Based on participating in teachers’ understanding, do they anticipate STEM applications to yield positive outcomes for their students’ 21st-century skills?

### 1.1. Cultivating Effective Sustained Teachers: The Imperative of STEM-Infused Professional Development

Recognizing the crucial role of teachers in solving complex problems, teacher professional development (PD) has emerged as a cornerstone of successful educational reform. Studies demonstrate the positive impact of effective PD programs on teacher quality, ultimately leading to improved student achievement and school culture (i.e., Garet et al. 2001). In addition, effective PD empowers teachers with deep domain knowledge, fosters continuous learning, broadens pedagogical repertoires, and promotes positive classroom behaviors (Orakci and Durnali 2023).

Despite policy efforts, integrating STEM disciplines effectively still faces challenges due to confusion and lack of clarity (Kelley and Knowles 2016). For example, transitioning from traditional methods to inquiry-based and project-oriented learning in STEM presents unique hurdles (Breiner et al. 2012). Many teachers lack the necessary pedagogical expertise and STEM content knowledge to implement these innovative approaches, leading to negative attitudes towards STEM education (Margot and Kettler 2019).

However, a good deal of research (i.e., Özbilen 2018) underscores that strategically designed STEM-infused PD programs offer solutions to these challenges. Furthermore, these programs equip teachers with the necessary knowledge and skills to shift negative attitudes towards enthusiasm and confidence in STEM (Özbilen 2018). They advocate for a transformative shift beyond mere integration, promoting holistic, STEM-based learning experiences that seamlessly connect disciplines and emphasize real-world problem-solving (Al Salami et al. 2017; Margot and Kettler 2019). As effective facilitators, teachers can confidently guide students through STEM complexities, fostering deeper understanding and nurturing learning environments (Al Salami et al. 2017; Margot and Kettler 2019).

On the other hand, as highlighted by leading scholars (i.e., Akbaşlı et al. 2020; Demir and Durnali 2022), we understand that teachers’ attitudes, self-efficacy, and problem-solving skills are very important for the successful implementation of STEM in the classroom. The bulk of the literature emphasizes that teachers have negative attitudes towards STEM. Then, professional development programs and in-service training practices can be a solution to eliminating teachers’ negative attitudes towards STEM. In other words, we know very well that teachers need skills beyond content delivery—interdisciplinary collaboration, creative problem-solving, and engaging learning environment design are crucial. In this regard, we can cultivate a generation of effective teachers who empower students to become capable citizens in a dynamic world through investing in high-quality hands-on professional development programs designed to cultivate positive teacher attitudes towards STEM, refine problem-solving abilities, and bolster self-efficacy in classroom implementation.

### 1.2. Cultivating Problem-Solvers: The Pedagogical Potential of STEM for Teachers

We know that rapid technological advancements, interconnected global challenges, and increasingly complex information environments necessitate this specific skillset. That said, the widely recognized importance of 21st-century skills pales in comparison to the urgent need for citizens equipped with specific higher-order thinking skills, particularly the ability to effectively navigate and solve complex real-world problems (Simamora et al. 2019). Said problem-solving proficiency transcends mere reactive responses; it necessitates a proactive, analytical approach to forging solutions in the face of unexpected challenges (Sen et al. 2018). Following this, equipping students with this indispensable skillset begins with fostering it within their teachers, highlighting the critical role of teacher development in shaping future generations of adept problem-solvers.

Recognizing the critical role of education in shaping future citizens, international assessments like PISA highlight the importance of measuring and nurturing students’ problem-solving capabilities (OECD 2014). In this endeavor, STEM emerges as a promising pedagogical framework. By integrating STEM into interdisciplinary learning experiences that address real-world problems, STEM fosters the development of critical thinking, creativity, collaboration, and, ultimately, problem-solving skills (National Science and Technology Council 2018).

However, the effectiveness of STEM in cultivating these skills hinges not only on curriculum design but also on the pedagogical expertise of teachers. Teachers themselves require robust problem-solving skills, coupled with the ability to create authentic problem scenarios and guide students through effective solution-seeking processes (Margot and Kettler 2019). STEM applications, wherein scientific inquiry ignites innovative engineering design solutions, provide a fertile ground for honing these crucial teacher competencies (Sen et al. 2018). Through active engagement with such practices, teachers can refine their ability to grapple with complex problems and translate their insights into engaging and effective learning experiences for their students.

Emerging research, exemplified by Huang et al. (2022) and Kutnick et al. (2022), underscores the compelling efficacy of professional development programs specifically designed to cultivate STEM-based problem-solving skills in teachers. This targeted investment empowers teachers to navigate the inherent complexities of problem-solving within the STEM context, fostering their transformation into transformative figures (Meijer et al. 2017; Reinius et al. 2022). By facilitating student engagement with authentic problem-solving scenarios, teachers equipped with these skills become instrumental in nurturing the development of essential 21st-century competencies, ultimately preparing their students to thrive as confident and capable citizens in the dynamic landscape of the present and future.

Furthermore, empirical studies make clear that practitioners often formatively transpose or simplify real-world tasks to suit classroom constraints. For example, Delahunty et al. (2021)—in a design-based evaluation of primary STEM programs in Ireland—reported teachers characterizing official curricula as more ideological than operational: they described tasks like CAD 3D-printing and biomimicry as “utopian,” too complex or misaligned with school routines to teach in authentic form, and said that teachers routinely “down-scaled” them to manageable scaffolds (Delahunty et al. 2021). A systematic analysis by Margot and Kettler (2019) similarly finds that many primary and middle school teachers transform engineering or inquiry lessons into heavily guided formats because limited preparation time and curricular rigidity make full integration impractical (Margot and Kettler 2019). Notably, professional development models such as the TRAILS program in the United States cultivate authenticity by asking teachers to solve real open-ended STEM problems themselves—e.g., designing biomimetic fishing lures and testing water-quality prototypes—and find that this experience produces a large gain (≈0.95) in STEM teaching self-efficacy, compared to control groups (Kelley et al. 2020). In our revised introduction, we now frame classroom “authenticity” in STEM as the teacher-mediated process of pedagogically translating complex real-world problems—rather than assuming unmediated transfer of real tasks to school settings.

### 1.3. The Enduring Influence of Teachers’ Self-Efficacy

The emergence of the construct of self-efficacy, as articulated by Bandura (1997), ranks as a landmark development in psychological understanding. Self-efficacy refers to teachers’ confidence in their ability to orchestrate and execute actions directed towards specific goals. In the educational domain, teacher self-efficacy emerges as a critical factor influencing teachers’ capacity to effectively implement pedagogical strategies and facilitate student learning outcomes (Skaalvik and Skaalvik 2007). It encompasses the belief that teachers possess the necessary skills to enact specific teaching behaviors that positively impact student achievement, motivation, and interest (Klassen and Tze 2014). Research underscores the potential of self-efficacy to not only shape teachers’ instructional practices but also influence their overall well-being (Avanzi et al. 2013) and their autonomy support, metacognition, and creative thinking (Orakci and Durnali 2023). Teachers with high self-efficacy tend to demonstrate increased job satisfaction, reduced professional burnout, and flourishing student relationships (Avanzi et al. 2013). Additionally, they exhibit stronger academic performance and the ability to cultivate positive connections with colleagues (Honicke and Broadbent 2016; Skaalvik and Skaalvik 2010).

The influence of self-efficacy extends beyond individual teachers, impacting the broader educational landscape. Notably, self-efficacy plays a pivotal role in fostering teachers’ willingness to engage in innovative pedagogical approaches such as STEM education (Margot and Kettler 2019). Pragmatically, effective implementation of STEM initiatives hinges on teachers possessing both adequate awareness and high self-efficacy in navigating the complexities of these interdisciplinary learning environments (Yaman and Aşılıoğlu 2022). Accordingly, professional development programs designed to support teachers in implementing STEM curricula have demonstrated efficacy in bolstering their confidence and self-efficacy in this domain.

Recent empirical work positions teacher self-efficacy in STEM as a domain-specific belief—that is, the confidence to orchestrate engineering design cycles, lead cross-disciplinary project work, and facilitate collective problem-solving in real classroom contexts (Menon et al. 2023). For example, a 2024 survey study of elementary teachers found that, while most believed that they “understood” STEM design processes in theory, the majority did not feel confident about implementing them until they received specialized STEM PD; they reported anxiety and perceived inadequacy as key reasons for their unwillingness to use such tasks in class (Buechel et al. 2024). For primary and middle school teachers, this framing contrasts sharply with general teaching efficacy: one can “understand” the STEM approach abstractly yet feel unprepared to guide students through an Iterative Prototyping Design Model, ask generative inquiry questions, or scaffold peer collaboration around real-world problems. Surveys of elementary teachers have repeatedly shown that many initially endorse STEM in principle but cite anxiety and perceived inadequacy as barriers to classroom enactment—barriers that diminish only after targeted, hands-on professional development (Gardner et al. 2019).

Several quasi-experimental interventions, including the TRAILS project for middle and secondary teachers, report effect sizes on STEM teaching self-efficacy between 0.46 and 0.95, with especially large gains when PD centers on authentic engineering design and inquiry tasks (Kelley et al. 2020). A systematic meta-analysis spanning 21 in-service STEM PD studies confirmed a strong overall impact (g = 0.64, *p* < .01), particularly when the training explicitly addressed STEM-aligned behaviors—such as guiding student prototyping teams, using iterative modeling, and introducing interdisciplinary collaboration in lessons (Zhou et al. 2023).

Beyond the content dimension, integrated models that foreground experiential learning (Kolb 1984) and cognitive development (Vygotsky and Cole 1978) have been shown to boost teachers’ self-efficacy via mastery, vicarious experience, and positive emotional arousal (Bandura 1997). For instance, professional development that blends open-ended design challenges with peer reflection and student feedback—such as Hong Kong’s air quality project using design thinking tasks—has been linked to measurable gains in teachers’ perceived competence and intrinsic motivation to teach STEM effectively (Chiu et al. 2021). Such scaffolded experiences closely align with STEM’s authentic epistemology and help teachers internalize self-efficacy in the specific practices of STEM education.

In the context of our study—evaluating a hands-on STEM training program with 30 in-service primary/middle school teachers, pre–post Likert measures of self-efficacy, attitudes, problem-solving skills, and follow-up interviews—the domain-specific efficacy approach helps tether theory to practice. It is no longer enough to conceptualize self-efficacy as general teaching confidence; rather, we operationalized it as confidence in enacting specific STEM-aligned pedagogical practices—for example, facilitating design thinking cycles, prompting interdisciplinary questions, or sustaining iterative prototyping challenges. This operationalization provides a clearer rationale for measuring pre and post changes aligned with policy uptake and classroom agency. In closing, taken together, the literature suggests that self-efficacy in STEM is a qualitatively different construct from general teacher efficacy. It is not merely confidence in teaching per se, but confidence in engaging in STEM-style pedagogy—design cycles, integration across subjects, handling ambiguity, and facilitating student-centered problem-solving.

### 1.4. Teachers’ Attitudes Towards STEM

We understand that STEM is an educational approach that integrates multiple disciplines into a unified learning experience (Bush et al. 2020). However, teachers from various disciplines typically receive training in a single disciplinary field during their undergraduate education. For teachers to effectively implement STEM activities in their classrooms, they must possess 21st-century skills that enable them to integrate knowledge from various disciplines (Şahin et al. 2024). Despite widespread implementation, expected performance outcomes have not been achieved in STEM-focused schools in the United States, primarily due to a lack of STEM-specific pedagogical content knowledge (Holdren et al. 2010). Contributing factors to this performance gap include deficiencies in interdisciplinary content knowledge, structural limitations, pedagogical shortcomings, and challenges in curricular integration (Holdren et al. 2010; Lo 2021; Margot and Kettler 2019; Rockland et al. 2010; Yaman and Aşılıoğlu 2022). Another critical issue is that many teachers are unaware of how STEM-related skills are applied within industry contexts (İnam 2020). These shortcomings negatively influence teachers’ perceptions and attitudes toward STEM (Wang et al. 2011) as well as their instructional strategies (Hill et al. 2020). Therefore, there is a pressing need to foster positive and proactive attitudes toward STEM among teachers (Al Salami et al. 2017). For STEM education to succeed, teachers must develop a high level of awareness and maintain favorable attitudes toward STEM (Yaman and Aşılıoğlu 2022).

Without a positive disposition, it is unlikely that teachers will transfer STEM knowledge to students in meaningful and actionable ways (Cortes et al. 2025). To overcome these challenges and cope with barriers in implementation, teachers require access to high-quality professional development programs (Derin et al. 2017; Lo 2021; Şahin et al. 2024). Without teacher motivation toward STEM, the full potential of STEM education cannot be realized (Maryna et al. 2025). Research shows that well-designed professional development programs positively impact teachers’ attitudes toward STEM (Erol and İvrendi 2025). Enhancements in teachers’ STEM competencies lead to more positive attitudes (Chiriacescu et al. 2023; Papagiannopoulou and Vaiopoulou 2024) and reductions in STEM-related anxiety (Amanova and Brynteson 2025). Teachers with favorable attitudes toward STEM tend to collaborate more with their colleagues and provide guidance in STEM implementation (Kalliontzi 2022). These experiences also contribute to the development of their pedagogical content knowledge (Glover et al. 2016). However, the existing literature on teachers’ attitudes toward STEM lacks consensus, indicating a need for further empirical investigation in this area (Cortes et al. 2025).

## 2. Methods

### 2.1. Research Design

By using a nested design, we aimed to explore comprehensively the effect of hands-on STEM training on teachers’ attitudes toward STEM, their self-efficacy in implementing STEM methodologies in the classroom, their problem-solving skills, their professional competence in integrating STEM applications into their instructional practices, and the effectiveness of these applications in enhancing students’ 21st-century skills. Before training, we first collected and analyzed quantitative data, adhering to the fundamentals of the nested design, to determine the level of teachers’ attitudes toward STEM, their self-efficacy in implementing STEM methodologies in the classroom and their problem-solving skills.

To triangulate the quantitative findings (Creswell 2019), qualitative data were collected and analyzed to examine how the intervention influenced teachers’ knowledge construction regarding professional competence in integrating STEM applications into their instructional practices, as well as the perceived effectiveness of these applications in fostering students’ 21st-century skills. Triangulation in this study was conducted at three levels:

(a) Within-method triangulation (quantitative): By using multiple validated instruments (SCISES, PSS, ATSE) targeting different but related constructs (self-efficacy, problem-solving, and attitudes);
(b) Within-method triangulation (qualitative): By ensuring the trustworthiness of the qualitative findings, semi-structured interviews were carried out with a selected subgroup of participants. The responses were analyzed through a dual-coding process, supported by inter-coder agreement techniques. To further enhance the study’s credibility and dependability, we followed Guba’s (1981) key trustworthiness criteria, including techniques such as triangulation and participant validation (credibility), maintaining detailed documentation of research procedures (dependability), and integrating triangulation with reflective journaling (confirmability). In line with Yin’s (2011) recommendations, the data collection and interpretation processes were conducted with methodological rigor to faithfully represent the investigated phenomena. The use of multiple data sources—including extended engagement with participants, interviews, observations, and structured questionnaires—enriched the data and supported the study’s overall validity (Arslan 2022). Furthermore, preliminary findings were shared with participants to verify accuracy and obtain feedback, thereby implementing member checking (Braun and Clarke 2013). Expert consultation across disciplines was also employed throughout the research process as an additional validation strategy (Creswell 2013). Finally, all interpretations and conclusions were systematically grounded in the empirical data to ensure coherence and evidential consistency.
(c) Across-method triangulation (between methods): By comparing and integrating results from both phases at the interpretation level to identify convergence, divergence, or complementarity between quantitative trends and teachers’ narrated experiences (Creswell and Clark 2007).

Furthermore, our study employed a single-group pre-test and post-test quasi-experimental design, allowing us to detect quantitatively and qualitatively significant changes in teachers’ competencies attributable to the intervention (Fraenkel et al. 2012; McMillan and Schumacher 2001). While the absence of a control group limits internal validity, the design is commonly used in educational settings (i.e., Menon et al. 2023; Kayaalp et al. 2025) to ethically assess interventions where random assignment is impractical, especially in professional development contexts (i.e., Gokbulut and Durnali 2023).

**Figure 1:**
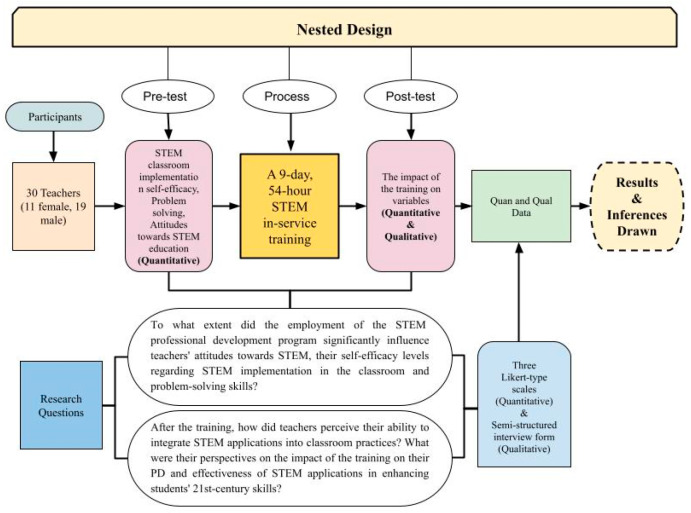
Research design. Adapted by the authors, drawing inspiration from the work of Kayaalp et al. (2025).

By employing this nested-methods approach, we were able to assess the extent to which the intervention influenced the targeted variables. The qualitative data enabled a deeper examination of the intervention’s impact, providing nuanced insights into teachers’ experiences and their subjective evaluations of its effectiveness. Ultimately, the integration of both quantitative and qualitative findings allowed for a more comprehensive interpretation, which was systematically discussed in the Results Section. From the outset, we recognized the value of a case study approach, which enabled an in-depth exploration of a specific unit—namely, a group of teachers—through diverse data collection methods, including interviews, observations, document analysis, and reports, within a defined timeframe (Creswell and Clark 2007). In this context, the case study delineates the duration of our STEM training program. Figure 1 provides a detailed representation of the research approach employed in the study.

### 2.2. The Participants

The participants consisted of teachers from Zonguldak Province’s public schools who attended the 2023 STEM in-service training program. The quantitative section involved thirty teachers (19 female, 11 male) selected through purposive sampling. In the qualitative section, twelve teachers (7 female, 5 male) were interviewed.

### 2.3. Data Collection, Tools and Interview Questions

Before and after the intervention, three measurement tools—SCISES, PSS, and ATSE (described below)—were used to assess the teachers’ pre- and post-intervention knowledge levels. In addition, following the training, a semi-structured interview was conducted. The structured interview questions are presented in the section titled “The qualitative section” below.

The quantitative section: To seek practically interpretable results, the quantitative study utilized a quasi-experimental pre- and post-test design. In doing so, we employed three measurement tools with established reliability, demonstrating reliable Cronbach’s Alpha coefficients for data collection.

STEM Classroom Implementation Self-Efficacy Scale (SCISES): The scale was developed by Yaman (2020), consisting of 3 factors and 23 items: Creating a Learning Environment (9 Items), STEM Integration (7 Items), and Establishing Real-Life Context (7 Items). The overall scale reliability coefficient obtained in this study was (Cronbach alpha) 0.85, and the sub-factors were 0.96, 0.95, and 0.43, respectively. This measure assesses teachers’ confidence in effectively integrating STEM activities and practices into their teaching.

Problem-Solving Scale (PSS): Adapted to Turkish by Coşkun (2004), the scale has a 5-point Likert structure and consists of 4 problem steps. There are 5 questions in each step and 20 questions in total. The Cronbach alpha reliability coefficient obtained was 0.94. This instrument evaluates teachers’ ability to approach and solve complex problems.

Attitudes Towards STEM Education Scale (ATSE): Developed by Yaman (2020), it consists of 17 items and is uni-factor. The reliability coefficient obtained was (Cronbach alpha) 0.98. This instrument gauges teachers’ overall perception of STEM’s value and impact on student learning.

The qualitative section: To gain a deeper understanding of the training program’s effectiveness and perceived value, the following semi-structured interview questions were posed to participating teachers on the final day of the intervention:

- Following the training, do you perceive yourself as capable of integrating STEM applications into your lessons or classroom practices? If so, please elaborate on the reasons behind your confidence.
- In your perspective, has the STEM program contributed to your professional development as a teacher? If so, how has it specifically impacted your practice?
- Based on your understanding, do you anticipate STEM applications to yield positive outcomes for your students’ 21st-century skills? Please explain your reasoning.

### 2.4. The STEM In-Service Training Program

This professional development program aimed to bolster the pedagogical practices of teachers in STEM disciplines, empowering them with the knowledge, skills, and strategies to cultivate engaging and impactful learning experiences for their students. The program adopted a blended approach, seamlessly integrating theoretical presentations with captivating hands-on activities and design challenges, fostering the development of essential 21st-century skills such as creative problem-solving and critical thinking. The intensive program spanned two weeks, delivering a comprehensive curriculum within a focused timeframe. Each day comprised six hours of dedicated learning activities, culminating in a total of 54 h of professional development. The program catered to a diverse group of 30 teachers, encompassing teachers from preschool, primary education, science, and mathematics branches. This inclusive approach ensured that the acquired knowledge and skills could be readily applied across various educational settings and age groups.

#### 2.4.1. The Training Content

- Foundations of STEM Education: The core principles and contemporary applications of STEM education through engaging presentations and discussions.
- Building the STEM Classroom: The practicalities of establishing a stimulating and well-equipped STEM learning environment, covering topics such as resource acquisition and laboratory setup.
- Developing STEM Expertise: Developing participants’ scientific knowledge and skills, focusing on key areas relevant to effective STEM instruction.
- Engaging Pedagogies: A range of research-based instructional models including the 5E Model, context-based learning, project-based learning, inquiry-based learning, and modeling, providing teachers with practical tools for fostering deep understanding and critical thinking in their students.
- Connecting STEM to the Real World: Contextualizing learning, enabling participants to effectively integrate STEM concepts into real-world scenarios and foster student interest and relevance.

#### 2.4.2. Training Schedule, Design, Teaching/Learning Activities, and Strategies

Day 1 and 2: Cultivating collaborative design thinking in STEM teachers

The opening day of the training program served to establish a baseline for participant knowledge and introduce key concepts related to STEM education and design thinking. This objective was achieved through a multifaceted approach, incorporating research-informed assessments, engaging activities, and informative presentations.

- Pre-test administration: Participants completed pre-tests designed to gather baseline data on their understanding of STEM principles and design thinking practices. These data will be instrumental in evaluating the program’s efficacy and identifying areas for future development.
- Collaborative tower building: Fostering teamwork and communication skills, a team-building activity challenged participants to construct the tallest tower using paper cups. This activity encouraged collaboration, problem-solving, and creative thinking within a competitive yet supportive environment.
- Demystifying STEM education: Participants engaged in an informative presentation covering the core objectives and significance of STEM education in contemporary pedagogical practices. This session highlighted the importance of integrating STEM principles into the curriculum to prepare students for future challenges and foster their critical thinking skills, as encouraged by some research (i.e., Baharin et al. 2018; Khalil and Osman 2017).
- Hands-on design thinking exploration: Transitioning from theory to practice, participants actively participated in a “Design Thinking Techniques” activity. Equipped with 5v DC motors, they collaborated to design and construct functional electric compasses. This hands-on experience provided a concrete understanding of the design thinking process, emphasizing iterative design, prototyping, and problem-solving through collaboration as encouraged by some research (i.e., Naghshbandi 2020; Palacin-Silva et al. 2017).
- Enhancing student motivation: The day concluded with a presentation focusing on “Student Motivation and Self-Confidence Enhancement Methods.” This session equipped participants with valuable strategies to cultivate a positive learning environment that fosters student engagement, intrinsic motivation, and self-confidence.

By combining these diverse elements, Day 1 laid a strong foundation for participants to delve deeper into design thinking principles and their application within STEM education throughout the following program days.

Day 3 and 4: Cultivating critical thinking through engineering design

Building upon the theoretical foundations established on Day 1 and 2, the third- and fourth-day training specifically targeted the cultivation of critical thinking skills within an engineering design context. The morning session commenced with presentations delving into the core tenets of the “Nature of Science,” emphasizing the significance of scientific literacy and the development of robust scientific process skills. This theoretical grounding was then seamlessly interwoven with the introduction of the “5E Instructional Model,” which provided a robust framework for fostering inquiry-based learning and deeper student engagement within STEM disciplines (Joswick and Hulings 2024; Kruatong et al. 2022).

To bridge the gap between theory and practice, the afternoon session transitioned to a dynamic, hands-on activity focused on “Force and Motion” concepts. Participants were challenged to design and construct functional vehicles, prompting them to actively apply the theoretical knowledge acquired earlier in the day. This engaging activity served as a springboard for critical thinking, as participants not only explored the intricate relationships between force, motion, and various STEM principles but also engaged in a reflective discussion aimed at critically analyzing their designs and identifying potential improvements. Those activities resonate with some scholars (i.e., Castillo Céspedes and Burgos Navarro 2022; Euler 2020). This fostered a growth mindset, encouraging participants to view challenges as opportunities for learning and refinement.

Day 5 and 6: Deepening pedagogical knowledge and application: Lesson planning and project-based learning through engineering design

Building upon the theoretical foundations established on Day 3 and 4, the program delved into practical applications of diverse pedagogical strategies. Presentations explored the structured approach of “5E Model-Based Planning,” equipping participants with tools for effective lesson design. Subsequent sessions delved into “Project-Based Learning” and “Inquiry-Based Learning” methodologies, highlighting their potential to stimulate student engagement and critical thinking within STEM contexts. To solidify theoretical understanding and translate it into tangible practice, participants engaged in a collaborative design challenge. Drawing inspiration from a provided sample problem, working groups collaboratively designed and tested functional wind turbine models, exemplifying the application of “STEM engineering products” within the presented pedagogical frameworks. These hands-on activities served as a bridge between theory and practice, as hinted by some research (i.e., Crippen and Antonenko 2018; Prins et al. 2016), fostering deeper understanding and the ability to translate the learned concepts into effective classroom strategies.

Day 7 and 8: Bridging mathematical concepts and STEM through contextualized learning

Shifting the focus toward seamless integration of mathematics within STEM activities, Day 7 and 8 delved into the theoretical underpinnings of “Mathematical Modeling,” exploring its diverse classifications and potent role in science education. Furthermore, the session highlighted the importance of “Context-Based Learning” in fostering meaningful understanding. To bridge theory and practice, participants engaged in collaborative learning, forming study groups tasked with developing a “fraction game.” As suggested by some research (i.e., Kertil and Gurel 2016), these activities served as a springboard for applying mathematical modeling concepts in a tangible and engaging manner, promoting active participation and creative problem-solving. The culmination of this activity involved group presentations showcasing the developed games, functioning as exemplary models for effectively integrating mathematics into STEM activities.

Day 9: Integration and evaluation

The last day of the workshop transitioned from theoretical foundations to practical application. To bridge the gap, carefully curated sample lesson plans, showcasing the seamless integration of STEM activities into diverse classroom settings, were presented. Following this exposure, facilitated discussions delved into tangible implementation strategies, addressing potential challenges and tailoring approaches to individual educational contexts. This interactive session empowered participants to translate theory into practice, ensuring that the acquired knowledge translated to impactful classroom experiences. To gauge the effectiveness of the program and assess participants’ knowledge acquisition, a formative assessment was administered. This culminating “final test” measured not only factual recall but also the ability to apply learned concepts and design effective STEM-infused lesson plans.

Beyond mere evaluation, the day concluded with a reflective summary of key takeaways. This final review not only reinforced important learnings but also solidified the program’s impact on participants’ professional development. The workshop’s comprehensive and engaging approach equipped teachers with valuable tools and strategies to seamlessly integrate STEM principles and practices into their curriculum, fostering a generation of innovative and creative learners through the cultivation of design thinking, critical thinking, and problem-solving skills.

**Figure 2:**
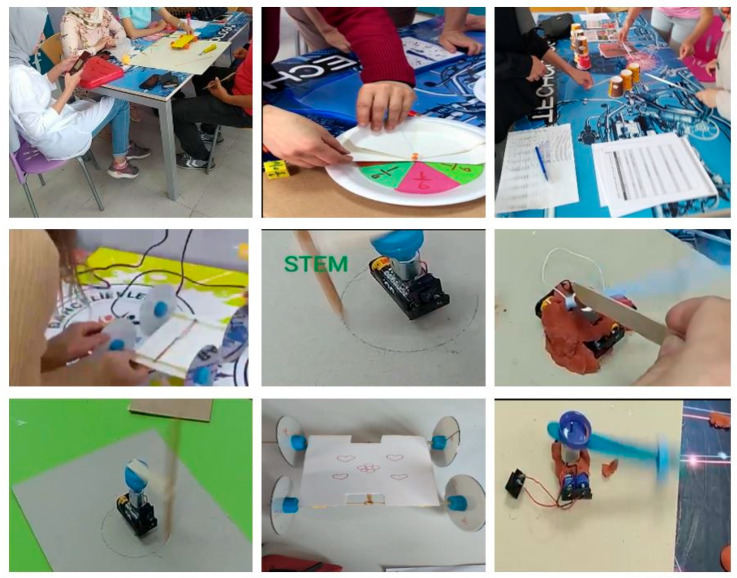
Images from the training (credit by the second author).

Figure 2 shows examples of applications from some activities made by teachers in the STEM training.

### 2.5. Ethical Considerations

The research adhered to stringent ethical guidelines, securing approval from the authors’ University Human Research Ethics Committee (report #174, dated 29 April 2022).

### 2.6. Data Analysis

Quantitative data: Following normality testing, which confirmed a normal distribution, paired samples t-tests were employed to analyze quantitative data. This method assessed potential changes in participants’ scores on the ATSE, SCISES, and PSS instruments before and after the STEM training.

Qualitative data: The qualitative data were analyzed using thematic analysis (Braun and Clarke 2013), following a structured multi-step process. First, all responses were transcribed and open-coded independently by two researchers. The codes were then discussed and refined through axial coding and grouped into broader themes. The coding process followed Miles and Huberman’s (1994) model of qualitative content analysis. To ensure reliability, intercoder agreement was calculated and exceeded 90%. That is, the reliability was calculated using Miles and Huberman’s formula (reliability = number of agreements/(total number of agreements + number of disagreements)). Direct quotes from teachers were integrated to enrich the qualitative findings and enhance authenticity. The themes were derived inductively and aligned with the three interview questions focusing on (1) teachers’ perceived ability to integrate STEM, (2) the impact of training on their professional development, and (3) anticipated benefits for students. Finally, the integration of qualitative and quantitative results occurred at the interpretation stage, where qualitative themes were used to deepen the explanation of observed quantitative changes and offer practical insight into the intervention’s effects. We resorted to the MAXQDA software during content analysis.

Confidentiality and anonymization: Throughout the research process, maintaining participant confidentiality was paramount. Teacher identities were anonymized, and teacher interview data were coded using pseudonyms (T1, T2, etc.).

## 3. Results

**Figure 3:**
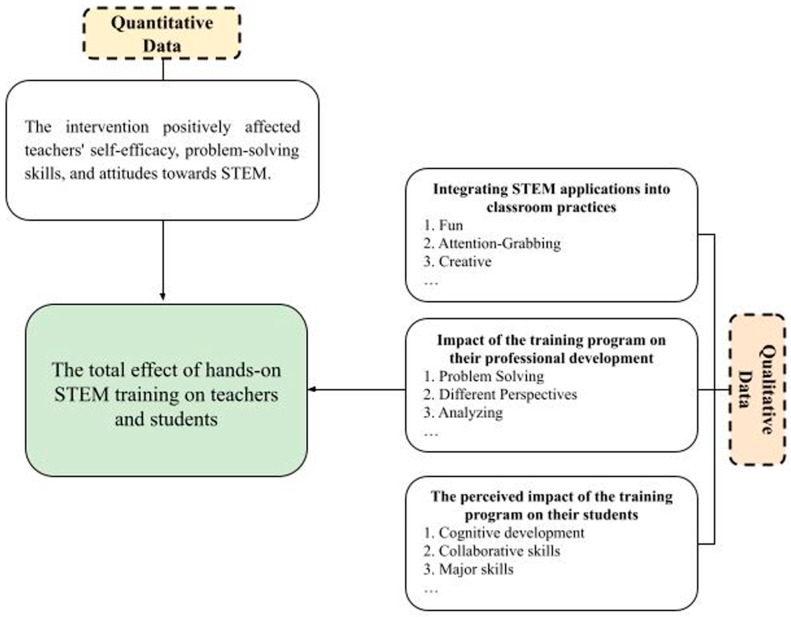
The qualitative findings complement the quantitative ones.

This section initially presents the quantitative findings, elucidating changes in teachers’ attitudes toward STEM education, their self-efficacy in implementing STEM in the classroom, and their problem-solving skills. These results were derived following the procedures of the nested design. Subsequently, the qualitative findings are introduced, focusing on the intervention’s role in enhancing teachers’ understanding of integrating STEM applications into their instructional practices, the impact of the training program on their professional development, and their perceptions of its influence on students. Consequently, the qualitative findings serve to complement and provide deeper insight into the interrelated quantitative results (Figure 3).

### 3.1. The Quantitative Findings on Teachers’ Attitudes Towards STEM Education, STEM Classroom Implementation Self-Efficacy and Problem-Solving Skills

This quantitative section presents the results of the paired samples *t*-test used to determine the significance of potential changes in teachers’ attitudes towards STEM education, self-efficacy in STEM classroom implementation, and problem-solving skills, as well as the mean pre-test and post-test scores. Additionally, we compared the mean pre-test and post-test scores for each measure to provide a descriptive overview of potential shifts in teachers’ understanding and skills. This approach aligns with the rigorous quantitative analysis and the exploration of factors influencing teachers’ professional development and instructional practices. By employing well-established statistical methods and clearly defining the constructions under investigation, the results presented below established a foundation for robust inferences and meaningful contributions to the field.

#### 3.1.1. The Teachers’ Level of Self-Efficacy in STEM Classroom Implementation Before and After the Training

Table 1 presents the results of *t*-tests conducted on pre- and post-intervention scores related to teachers’ self-efficacy in STEM classroom implementation. This analysis aims to elucidate the impact of the training program on teachers’ confidence in integrating STEM practices into their instruction.

Table 1 presents that there was a statistically significant (*p* < .05) difference between the arithmetic mean scores of the creating a learning environment factor pre-test (M = 4.08) and post-test (M = 4.57), STEM Integration factor pre-test (M = 3.77) and post-test (M = 4.51), establishing real-world context factor pre-test (M = 3.97) and post-test (M = 4.84), and SCISES (Total) pre-test (M = 3.95) and post-test (M = 4.63). The significant difference was in favor of the post-test in SCISES total and all sub-factors. In other words, the results of the paired samples t-tests indicated that there were significant increases in teachers’ self-efficacy across all three factors of STEM classroom implementation: creating a learning environment, STEM integration, and establishing real-world context. These results suggest that the training program was effective in improving teachers’ confidence in their ability to integrate STEM activities and practices into their teaching. These results suggest that the training program was effective in improving teachers’ confidence in all three aspects of STEM classroom implementation. This is important because high teacher self-efficacy is crucial for adopting new practices, fostering student success, and building strong professional relationships. It improves instructional methods, boosts well-being, and enhances autonomy, metacognition, and creative thinking (Avanzi et al. 2013; Orakci and Durnali 2023).

#### 3.1.2. The Teachers’ Level of Problem-Solving Skills Before and After the Training

With Table 2, we delve into the effectiveness of the training program for enhancing teachers’ problem-solving capabilities. Paired samples t-tests are employed to compare pre- and post-intervention scores on a validated instrument measuring problem-solving skills. This rigorous analysis elucidates whether the program successfully equipped teachers with the necessary cognitive tools to approach and effectively solve complex problems.

Table 2 shows that there is a statistically significant (*p* < .05) difference between the arithmetic mean scores of the P1 factor pre-test (M = 4.44) and post-test (M = 4.66), P2 factor pre-test (M = 4.46) and post-test (M = 4.72), P3 factor pre-test (M = 4.38) and post-test (M = 4.66), P4 factor pre-test (M = 4.42) and post-test (M = 4.76), and P_Total pre-test (M = 4.43) and post-test (M = 4.70). The significant difference is in favor of the post-test in all of the P_Total and sub-factors. These statistically significant increases across all dimensions favor the post-test, providing robust support for the intervention’s effectiveness in improving teachers’ abilities to approach and solve complex problems. The findings present compelling evidence that the targeted training successfully equipped teachers with enhanced problem-solving skills, potentially impacting their instructional practices and fostering student learning.

#### 3.1.3. The Teachers’ Attitudes Towards STEM Education Before and After the Training

The *t*-test results of the pre- and post-test scores of the teachers regarding their level of attitudes towards STEM education before and after the training are given in Table 3.

Table 3 shows that the arithmetic mean scores for the ATSE pre-test (M = 4.14) and post-test (M = 4.50) do not exhibit a statistically significant difference (*p* > .05). This suggests that, while there is a slight increase in the mean score from pre-test to post-test, this increase cannot be attributed to the intervention with any degree of certainty. That is, we generally considered the *p*-value of .05 the threshold for statistical significance. This means that there is a less than 5% chance that the observed difference in mean scores could have occurred by chance. In this case, the *p*-value is greater than .05, indicating that the observed difference could be attributed to chance with a probability greater than 5%. It is important to note that a lack of statistical significance does not necessarily mean that the training had no effect. It is possible that the training had an effect that could be seen explicitly from arithmetic mean scores. Additionally, other factors, such as the participants’ motivation or prior knowledge, may have influenced the results.

### 3.2. Qualitative Exploration of Teacher Experiences

Building upon the quantitative analysis, this section delves into the lived experiences of teachers who participated in the STEM training program. Through thematic analysis of their diverse perspectives gathered via semi-structured interviews, we aimed to illuminate their perceived effectiveness of the program and potential shifts in their professional development and instructional practices. This qualitative approach complements the quantitative findings and aligns with the exploring factors influencing teacher development and pedagogical practices.

#### 3.2.1. The Views of Teachers on Whether the Training They Attended Increased Their Capability of Integrating STEM Applications into Their Lessons or Classroom Practices and the Reasons Behind Their Confidence

**Figure 4:**
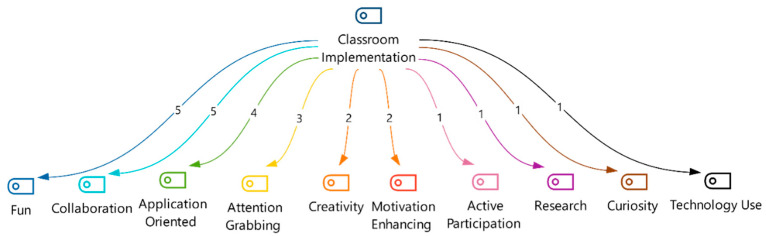
Max map codes for the theme of STEM in-class implementation.

Following the professional development program focused on STEM integration, qualitative analysis investigated teachers’ responses to the following open-ended question: “Do you believe you can utilize STEM applications in your lessons or classroom activities? Why?” This inquiry aimed to explore their perceived ability and rationale for implementing STEM practices after the training. Thematic analysis-generated codes, with those pertaining specifically to “In-Class Application”, are presented in Figure 4.

Figure 4 presents ten salient codes extracted from the qualitative analysis of teacher interviews. Notably, the identified codes and their frequency of mention offer valuable insights into key priorities and perceived benefits associated with STEM education implementation.

Engagement and enjoyment (fun, N = 5; attention-grabbing, N = 3, creative, N = 2): The paramount importance of fostering student engagement and enjoyment through intrinsically motivating activities emerges as a prominent theme. Teachers emphasize the effectiveness of STEM applications in “drawing the student’s attention to the lesson…and motivating the student” (T1, T2, T8, T11) through fun, creativity, and captivating activities.

Collaboration (N = 5): Recognition of the value of collaborative learning environments in facilitating problem-solving and peer-to-peer learning is evident. Teachers underscore the potential of integrating lesson outcomes with STEM to facilitate “collaboration work” (T2, T5).

Real-world application (application-oriented, N = 4; research, N = 1): Integrating STEM concepts with authentic, real-world scenarios is perceived as crucial for enhancing relevance and student motivation. Teachers highlight their ability to “prepare lesson activities using simple real-world materials in the classroom” (T4), emphasizing the potential of such practices to increase engagement and understanding.

Motivation and curiosity (motivation enhancing, N = 2; active participation, N = 2; curiosity, N = 1): Cultivating both intrinsic motivation and curiosity towards STEM subjects in both teachers and students is identified as a critical objective. Quotes highlighting the value of STEM in preparing “more qualified eTwinning projects” and leveraging its overlap with science and math to “contribute to the student’s success in upper grades” (T2, T4, T5, T7) exemplify this theme.

Technology integration (technology use, N = 1): While technology use was mentioned less frequently, its potential to support effective STEM learning is acknowledged. Teachers recognize the potential of technology, focusing its ability to “make applications with simple tools and materials” (T1).

This thematic analysis revealed that teachers perceived STEM education as a transformative tool for creating engaging and student-centered learning environments. The identified themes underscored the importance of fostering fun, collaboration, real-world relevance, and intrinsic motivation in student learning. While technology integration emerged with less emphasis, its potential as a learning tool remains recognized. Incorporating these perspectives into the design and implementation of STEM education programs can optimize their positive impact on student engagement, knowledge acquisition, and future career preparedness.

#### 3.2.2. Exploring the Range of Views Expressed by Teachers Regarding the Perceived Impact of the Training Program on Their Professional Development

**Figure 5:**
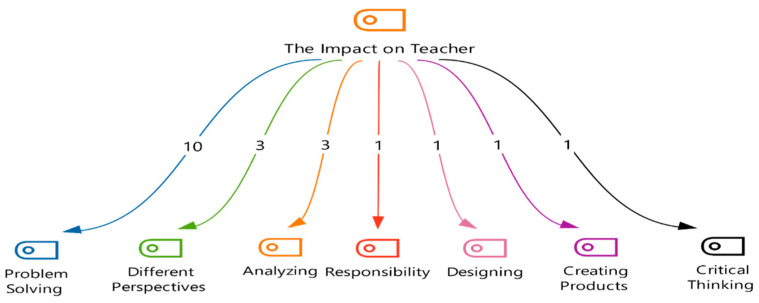
Max map codes for “The Impact on Teacher” theme.

Following participation in a STEM-focused training program, participants engaged in a reflective activity, responding to the following prompt: “Did STEM education contribute to your professional development? How?” A robust qualitative analysis was conducted on their responses, generating thematic codes. Figure 5 delves into the “Impact on the Teacher” theme, illuminating how participants perceived the training’s influence on their professional growth and pedagogical practices.

Figure 5 highlights the seven codes gathered from thematic analysis results related to teachers’ perceived impact of STEM education. “Problem-Solving” emerged as the most prominent theme (N = 10), followed by “Different Perspectives” and “Analyzing” (N = 3 each). Other recurring themes included “Responsibility”, “Designing”, “Creating Products”, and “Critical Thinking” (N = 1 each).

Qualitative analysis further elucidates these themes. Teachers attributed improvements in problem-solving (T1, T2, T3, T4, T5, T6, T7, T8, T9, and T11), analyzing (T5, T8, and T9), and critical thinking skills (T1, T2, and T11) to the STEM training. Participants reported enhanced abilities to create problem-based solutions (T1, T5, T10, and T11), develop products, and revise their creations (T5). The training was perceived as fostering skills applicable to real-world problem-solving (T3, T5). Moreover, teachers highlighted the value of questioning (“Why?” and “How to improve?”) for effective problem-solving (T4). Finally, one participant emphasized the synergy between in-class and out-of-class activities in facilitating the transfer of problem-solving skills from school to everyday life (T5).

#### 3.2.3. Exploring the Range of Views Expressed by Teachers Regarding the Perceived Impact of the Training Program on Their Students

**Figure 6:**
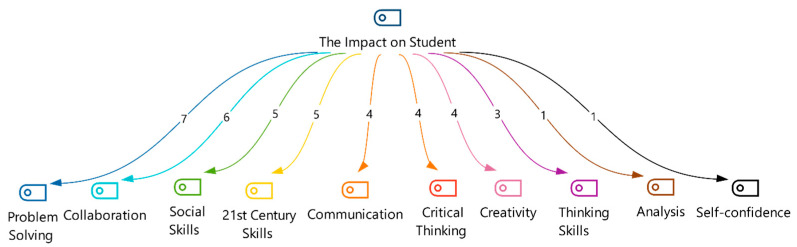
Max map codes for “The Impact on Student” theme.

Following the training program, qualitative analysis of teachers’ responses to “Do you think STEM applications will have contributions to students? Why?” revealed a prominent theme: impact on the student. Figure 6 presents the identified codes within this theme.

Figure 6 presents themes derived from thematic analysis of teacher narratives, exploring their perceived impact of STEM education on student development. Ten key codes emerged:

- Cognitive development: Emphasis on problem-solving (N = 7), critical thinking (N = 4), and analysis (N = 1),
- Collaborative skills: Collaboration (N = 6) and communication (N = 4),
- Major skills: 21st-century skills (N = 5), creativity (N = 4) and thinking skills (N = 3),
- Social–emotional learning: Self-confidence (N = 1) and social skills (N = 5).

Teachers elaborated on these themes, describing students “learning by doing” through problem identification, collaboration, design, and communication (T1, T2, and T4). This process was perceived to foster critical thinking, problem-solving, creativity, and collaboration (T1, T2, T4, T5, and T11). Additionally, teachers emphasized the development of self-confidence, communication, and leadership skills within group work (T1, T5, and T9). Participant quotes (denoted as “T” followed by a number) underscored the perceived contributions of STEM to 21st-century skill development, career preparation, and overall student well-being (T5, T6, T7, T8, T11, and T12).

## 4. Discussion

This study aimed to evaluate the impact of a STEM-focused professional development program on teachers’ self-efficacy, problem-solving skills, and attitudes toward STEM education. The findings demonstrated statistically significant improvements in teachers’ self-efficacy in implementing STEM practices and in their problem-solving skills across all measured dimensions. However, changes in attitudes toward STEM education, while slightly positive, were not statistically significant. The qualitative analysis further revealed that teachers perceived improvements in their instructional integration of STEM, professional competence, and their students’ cognitive and social development. These findings, interpreted in light of existing research, underscore the nuanced role of targeted PD in shaping educational practice and outcomes.

One of the most robust outcomes of this study was the marked improvement in teachers’ self-efficacy in implementing STEM practices. Participants reported heightened confidence in their ability to design and facilitate STEM activities, foster interdisciplinary learning, and create supportive learning environments—domains where statistically significant growth was observed. These findings align with a growing body of literature suggesting that well-structured professional development directly enhances teachers’ beliefs in their instructional competence (Altaş 2018; Yaman 2020; Yaman and Aşılıoğlu 2022). Specifically, Altaş (2018) and Yaman and Aşılıoğlu (2022) reported significant gains in the “Learning Environment Creation” sub-dimension of self-efficacy, which mirrors our quantitative findings. This sub-dimension encompasses teachers’ confidence in organizing student-centered, inquiry-driven learning contexts essential for effective STEM education.

The observed self-efficacy gains are particularly noteworthy given the short duration of the training. While these results demonstrate the capacity of intensive PD to generate immediate benefits, other studies raise important considerations regarding sustainability. For instance, Yaman (2020) emphasized that longer or ongoing PD opportunities tend to produce more sustained changes in teacher beliefs and practices. Similarly, Geng et al. (2019) reported that even STEM-experienced teachers exhibited initial resistance or skepticism, underscoring the need for prolonged engagement and follow-up support to foster deeper attitudinal and pedagogical transformation.

Another significant outcome of the training program was the enhancement of teachers’ problem-solving skills, observed across all dimensions—pre-problem strategy, in-process behavior, post-problem reflection, and overall methodology. These improvements are in line with the literature that stresses the importance of exposing teachers to a variety of strategies to strengthen their analytical and reflective capacities (Aygen 2018). Our findings extend the current evidence by illustrating how systematic PD can bolster problem-solving not just in theoretical terms, but in real-world instructional contexts.

Notably, the qualitative data illuminated how teachers began to apply problem-solving frameworks not only to their own teaching practices but also to student learning processes. This supports Yaman and Aşılıoğlu’s (2022) assertion that teacher development initiatives have a trickle-down effect, ultimately shaping classroom environments and influencing student outcomes. Furthermore, our findings confirm Hacıoğlu et al.’s (2017) conclusion that PD can mitigate common instructional challenges such as time constraints, classroom management, and lesson planning. Teachers in our study reported that the training equipped them with strategies to more efficiently organize their time, sequence activities, and manage student interactions, enabling more fluid implementation of STEM tasks.

While self-efficacy and problem-solving exhibited notable progress, the lack of statistically significant change in attitudes toward STEM education reveals an area of concern. Although qualitative reflections suggested slightly more positive dispositions, these were not strong enough to reflect in the quantitative scores. This result suggests that short-term interventions may be insufficient to influence deeper belief systems. This finding is corroborated by Yaman (2020), who contends that extended exposure and iterative learning are critical for altering long-standing attitudes and pedagogical orientations.

Moreover, Geng et al. (2019) highlighted that even in contexts where STEM has been integrated for several years, teacher attitudes remain resistant to rapid change, particularly in areas requiring substantial cognitive and emotional investment. Thus, while skill-based competencies can be relatively quickly enhanced, shifting mindsets necessitates a more prolonged and holistic approach.

The qualitative analysis revealed several themes supporting and enriching the quantitative findings. A salient theme was teachers’ improved ability to integrate STEM concepts into their instructional designs. Teachers emphasized the value of real-world application, highlighting how authentic scenarios made STEM content more engaging and meaningful for students. This finding aligns with Avery and Reeve (2013), who argue that professional development should bridge theoretical standards with practical, everyday experiences to foster student engagement and learning.

Another critical theme was collaboration. Teachers frequently mentioned peer interaction and teamwork as valuable aspects of the training, not only for their own learning but also as a pedagogical strategy they intended to implement. This echoes the work of Marx et al. (1997) and Maskit (2011), who identified collaboration as essential in supporting teachers to adopt and adapt new instructional approaches.

Additionally, the training appeared to foster teachers’ understanding of the motivational aspects of STEM instruction. Teachers noted increased student curiosity, creativity, and enjoyment—factors identified by Capraro et al. (2016) as essential for effective STEM education. These motivational gains contribute to a learning environment where students are more likely to engage deeply, take intellectual risks, and persist through challenges—core characteristics of STEM-oriented learning.

Teachers also identified numerous 21st-century skills that were supported through the training. These included creativity, critical thinking, communication, leadership, and collaborative problem-solving. Our findings parallel those of Polgampala et al. (2017) who emphasized the role of STEM education in developing transversal competencies that are essential for success in modern learning and work environments. In particular, critical thinking and design thinking emerged as frequently mentioned benefits, which supports Teo and Ke’s (2014) and Capraro et al.’s (2016) assertion that STEM offers fertile ground for higher-order cognitive development.

An equally compelling outcome of the PD program was its perceived impact on students’ social–emotional learning. Teachers reported that STEM activities enhanced student self-confidence, promoted teamwork, and supported a sense of belonging—findings that resonate with Affouneh et al. (2020), who underscored the connection between teacher competence and student socio-emotional outcomes. While our study did not measure these dimensions quantitatively, the qualitative data point to meaningful affective and interpersonal benefits, which may merit further investigation in future research.

While the importance of technology in STEM education is widely acknowledged, its integration appeared to be one of the less developed themes in our data. Teachers recognized the value of digital tools but expressed a need for more targeted training in effectively incorporating technology into instruction. This finding supports Capraro et al. (2016), who called for PD programs that go beyond content delivery and support meaningful, pedagogically sound technology integration. It is clear from our results that future PD efforts should allocate more time and resources to this area, particularly in light of increasing digital transformation in education (Durnali 2025). We certainly understand that this convergence of the value of digital tools—generative artificial intelligence, simulations, virtual reality, animations, mobility, connectivity, and application ecosystems— situates technology at the core of digital transformation in STEM education and teaching practices, representing a critical aspect of the technology integration paradigm (i.e., Durnali 2022; Durnali et al. 2019, 2025; Durnali and Çoban 2025; Durnali and Limon 2020) in STEM education. Finally, our findings, while resonant with international studies, also underscore the importance of contextualizing PD to local needs. For example, Yaman and Aşılıoğlu (2022) emphasized region-specific nuances in the Turkish context, while Geng et al. (2019) and Hsu et al. (2011) discussed broader, global challenges such as teacher workload, training sustainability, and curricular alignment. Our study adds to this dialog by highlighting how short, structured PD interventions can be impactful but must be tailored to cultural and systemic conditions to achieve lasting success.

In closing, to address this important distinction, it is worth emphasizing that while the quantitative analyses in this study demonstrate measurable improvements in teachers’ self-efficacy and problem-solving performance, they cannot, on their own, fully establish causality regarding the intervention’s effects, particularly given the relatively small sample size. In this respect, the qualitative findings serve as a critical complement by providing teachers’ lived experiences and reflective accounts that directly attribute perceived changes in their practices and student outcomes to the professional development program. This dual perspective not only strengthens the credibility of the results but also acknowledges the inherent limitations of statistical inference in small-n studies, thereby underscoring the value of integrating qualitative feedback to more fully capture the scope and impact of the intervention.

## 5. Conclusions, Limitations, and Recommendations

While our study demonstrated the intervention’s effectiveness, exploring its long-term impact on classroom practices and student learning outcomes would be valuable. Additionally, investigating the specific training components that contributed most to improvement could inform future program development. Furthermore, examining the influence of school climate and individual teacher characteristics, as emphasized by Thibaut et al. (2018a) and Al Salami et al. (2017), could provide deeper insights into the intervention’s generalizability and potential moderators of its effectiveness.

Our study aimed to assess the impact of a STEM education intervention on both teachers’ attitudes towards STEM. Although the results did not reveal statistically significant positive effects, this finding offers valuable insights and prompts further discussion within the context of the existing literature. Our findings resonate with past research highlighting the limitations of undergraduate STEM education for teachers and the potential of targeted professional development programs to improve attitudes. However, the lack of observed change in this study suggests the specific program might not have addressed existing knowledge gaps or provided meaningful application strategies. Aygen (2018) emphasizes the challenge of effectively integrating engineering design into STEM education. The intervention in this study may not have adequately addressed this, limiting its impact on both teacher attitudes and student learning. Al Salami et al. (2017) suggest that witnessing positive student outcomes can boost teacher attitudes towards new approaches.

The null results raise questions about whether teachers perceived the intervention as demonstrably effective on student learning, potentially hindering positive attitude shifts. However, the null results necessitate further investigation into the intervention’s design, implementation fidelity, and potential confounding factors. While the study produced null results, its significance lies in highlighting the need for further exploration and refinement of STEM education interventions. By considering the limitations of this study and drawing connections to the existing literature, we can pave the way for future research that leads to more effective programs for preparing teachers and enhancing student learning in STEM fields.

In closing, our study investigated the impact of a professional development program in STEM education on teachers’ self-efficacy, problem-solving skills, and attitudes towards STEM. The findings provide significant insights. That is, the intervention positively impacted teachers’ self-efficacy in implementing STEM practices, aligning with existing research demonstrating the effectiveness of professional development in this area. The program significantly enhanced teachers’ problem-solving abilities across various dimensions, addressing challenges identified in previous studies and surpassing existing research by potentially equipping teachers to cultivate effective problem-solving environments for students. Although no statistically significant changes were observed in attitudes towards STEM, this null result is valuable in highlighting the need for further exploration and refinement of STEM education interventions.

Our study acknowledges several limitations. That is, the study assessed immediate post-intervention effects, but the long-term impact on classroom practices and student learning remains unexplored. The influence of school climate and individual teacher characteristics was not explored, potentially limiting generalizability. Building on these limitations, future research should investigate the long-term impact of the intervention on classroom practices and student learning outcomes. They can examine the influence of school climate and individual teacher characteristics on intervention effectiveness. Moreover, the findings of our study are based on the perspectives of 30 teachers from public schools in Turkey, which may restrict the generalizability of the results to broader populations or different educational contexts. Another limitation of the study is its reliance on a single-group pre-test and post-test design, even if our qualitative findings provided valuable insights that aided in the interpretation of the quantitative results. That said, our research highlights the necessity for further investigation into the effectiveness of STEM training across diverse educational settings and among teachers with varying backgrounds.

In light of these findings and limitations, an explicit area for future research involves investigating the sustainability of professional development impacts over time, particularly whether gains in self-efficacy and problem-solving translate into long-term changes in teaching practices and student outcomes. Future studies should also disentangle which specific elements of STEM-focused professional development—such as collaborative design, technology integration, generative AI implementation, or real-world application—are most influential in shaping teachers’ beliefs and practices. Moreover, cross-contextual comparisons across different regions and school cultures would provide valuable insights into how systemic and cultural factors moderate effectiveness. By pursuing these directions, researchers can build a more comprehensive understanding of how professional development in STEM education can be refined, scaled, and sustained to maximize both teacher growth and student success.

## Figures and Tables

**Table 1:** The *t*-test results of the pre- and post-test scores of the teachers regarding their level of self-efficacy in STEM classroom implementation.

Scale	Test	N	M	Sd	*t*	*p*
Creating a learning environment	Pre-test Post-test	30	4.08	0.78	−3.46	0.00 *
		30	4.57	0.40		
STEM integration	Pre-test Post-test	30 30	3.77 4.51	0.85 0.47	−4.88	0.00 *
Establishing real-world context	Pre-test Post-test	30 30	3.97 4.84	0.85 1.42	−2.79	0.00 *
SCISES Total	Pre-test Post-test	30 30	3.95 4.63	0.78 0.59	−4.12	0.00 *

* *p* < 0.01.

**Table 2:** The *t*-test results of the pre- and post-test scores of the teachers regarding their level of problem-solving skills.

	Test	N	M	Sd	*t*	*p*
P1	Pre-test Post-test	30	4.44	0.62	−2.13	0.04 *
		30	4.66	0.41		
P2	Pre-test Post-test	30 30	4.46 4.72	0.50 0.39	−2.59	0.01 *
P3	Pre-test Post-test	30 30	4.38 4.66	0.56 0.51	−2.35	0.02 *
P4	Pre-test Post-test	30 30	4.42 4.76	0.46 0.38	−3.30	0.00 *
P_Total	Pre-test Post-test	30 30	4.43 4.70	0.44 0.37	−3.20	0.00 *

* *p* < 0.01 P1: What do you do before you start solving a difficult problem? P2: What do you do while you are solving the problem? P3: What do you do after you have finished solving the problem? P4: What method do you use to solve the problem?

**Table 3:** The *t*-test results of the pre- and post-test scores of the teachers regarding their level of attitudes towards STEM education.

	Test	N	M	Sd	*t*	*p*
ATSE	Pre-test Post-test	30	4.14	0.97	−1.64	0.11
		30	4.50	1.03		

## Data Availability

The raw data supporting the conclusions of this article will be made available by the authors on request.
